# Supplementary material for: The Role of FBXW7 in Gynecologic Malignancies
Source: Cells. 2023 May 17;12(10):1415. doi: 10.3390/cells12101415 (PMC10216672; doi:10.3390/cells12101415)
Supplement: Supplementary file 1 [file cells-12-01415-s001.zip › cells-2329930-supplementary.pdf]

Supplementary Table S1. Summary of the study conducted using the cBioPortal Database*

Study	N. of cases	N. of cases with *FBXW7* mutation detected	Frequency (%)
Cervical Squamous Cell Carcinoma (TCGA, PanCancer Atlas)	297	39	13.13
Endometrial Carcinoma (CPTAC, Cell 2020)	81	15	18.52
Endometrial Carcinoma MSI (MSK, Clin Cancer Res 2022)	181	44	24.31
Endometrial Carcinoma cfDNA (MSK, Clin Cancer Res 2022)	44	12	27.27
Low-Grade Serous Ovarian Cancer (MSK, Clin Cancer Res 2022)	119	not profiled	--
Ovarian Serous Cystadenocarcinoma (TCGA, PanCancer Atlas)	585	17	2.91
Small Cell Carcinoma of the Ovary (MSK, Nat Genet 2014)	12	not profiled	--
Squamous Cell Carcinoma of the Vulva (CUK, Exp Mol Med 2018)	15	2	13.33
Uterine Carcinosarcoma (Johns Hopkins, Nat Commun 2014)	22	5	22.73
Uterine Carcinosarcoma (TCGA, PanCancer Atlas)	57	22	38.60
Uterine Clear Cell Carcinoma (NIH, Cancer 2017)	16	1	6.25
Uterine Corpus Endometrial Carcinoma (TCGA, PanCancer Atlas)	529	98	18.53

*12 studies that are manually curated, including TCGA and non-TCGA studies with no overlapping samples. cBioPortal Database (https://www.cbioportal.org/).
